# Transcranial direct-current stimulation increases extracellular dopamine levels in the rat striatum

**DOI:** 10.3389/fnsys.2013.00006

**Published:** 2013-04-11

**Authors:** Tomoko Tanaka, Yuji Takano, Satoshi Tanaka, Naoyuki Hironaka, Kazuto Kobayashi, Takashi Hanakawa, Katsumi Watanabe, Manabu Honda

**Affiliations:** ^1^Department of Functional Brain Research, National Institute of Neuroscience, National Center of Neurology and PsychiatryTokyo, Japan; ^2^Human Information Science Laboratory, NTT Communication Science LaboratoriesKanagawa, Japan; ^3^CREST, Japan Science and Technology AgencySaitama, Japan; ^4^Center for Fostering Young and Innovative Researchers, Nagoya Institute of TechnologyAichi, Japan; ^5^Department of Molecular Genetics, Institute of Biomedical Sicence, Fukushima Medical University School of MedicineFukushima, Japan; ^6^Integrative Brain Imaging Center, National Center of Neurology and PsychiatryTokyo, Japan; ^7^Cognitive Science Laboratory, University of TokyoTokyo, Japan

**Keywords:** basal ganglia, dopamine, Parkinson disease, transcranial direct current stimulation, striatum

## Abstract

**Background:** Transcranial direct-current stimulation (tDCS) is a non-invasive procedure that achieves polarity-dependent modulation of neuronal membrane potentials. It has recently been used as a functional intervention technique for the treatment of psychiatric and neurological diseases; however, its neuronal mechanisms have not been fully investigated *in vivo*.

**Objective/Hypothesis:** To investigate whether the application of cathodal or anodal tDCS affects extracellular dopamine and serotonin levels in the rat striatum.

**Methods:** Stimulation and *in vivo* microdialysis were carried out under urethane anesthesia, and microdialysis probes were slowly inserted into the striatum. After the collection of baseline fractions in the rat striatum, cathodal or anodal tDCS was applied continuously for 10 min with a current intensity of 800 μA from an electrode placed on the skin of the scalp. Dialysis samples were collected every 10 min until at least 400 min after the onset of stimulation.

**Results:** Following the application of cathodal, but not anodal, tDCS for 10 min, extracellular dopamine levels increased for more than 400 min in the striatum. There were no significant changes in extracellular serotonin levels.

**Conclusion:** These findings suggest that tDCS has a direct and/or indirect effect on the dopaminergic system in the rat basal ganglia.

## Introduction

Transcranial direct-current stimulation (tDCS) is a non-invasive technique in which a weak DC is used to polarize target brain regions (Nitsche and Paulus, 2000). Several studies have previously shown that tDCS affects motor function and learning in healthy subjects, presumably by changing the neuronal activity of the stimulated site (Wassermann and Grafman, 2005; Tanaka et al., 2009; Bachmann et al., 2010; Fox, 2011; Schambra et al., 2011). It is also effective in patients with psychiatric and neurological diseases, and so has the potential to be used as an adjuvant strategy in the rehabilitation of motor and cognitive deficits caused by neurological disorders (Hummel et al., 2005; Boggio et al., 2006; Fregni et al., 2006; Lefaucheur, 2009; Murphy et al., 2009; Nitsche et al., 2009; Benninger et al., 2010; Tanaka et al., 2011a; Brunoni et al., 2012). In addition, some *in vivo* animal studies have investigated the behavioral and biological effects of tDCS (Kim et al., 2010; Wachter et al., 2011; Laste et al., 2012). Nevertheless, the mechanisms underlying tDCS are largely unknown, particularly with regard to its effects on the neuronal network.

tDCS directly modulates neuronal membrane potentials beneath the stimulus electrode. However, it might also have a remote or systematic effect on the neuronal circuit. Indeed, increasing evidence from human electrophysiological and neuroimaging studies suggests that tDCS modulates brain activities in cortical or subcortical areas other than the stimulated site (Lang et al., 2005; Bachmann et al., 2010; Antal et al., 2011; Binkofski et al., 2011; Halko et al., 2011), possibly via anatomical connections (Veening et al., 1980; Selemon and Goldman-Rakic, 1985; McGeorge and Faull, 1989). Recently, human experiments using fMRI reported that tDCS can also modulate resting-state functional connectivity in distinct functional networks of the brain (Keeser et al., 2011). Furthermore, it has been demonstrated that an increase of phosphorylation of trkB, which is a receptor of Brain-derived neurotrophic factor (BDNF), was induced by DCS *in vitro* (Fritsch et al., 2010). These comprehensive effects through neuronal networks may be observable at the neurotransmitter level, as well as at the electrophysiological and metabolic levels. Previously, pharmacological approaches based on human drug intake have suggested the involvement of glutamatergic, γ-aminobutyric acid (GABA)-ergic, and dopaminergic systems in long-term tDCS effects (Liebetanz et al., 2002; Nitsche et al., 2003). These experiments, however, reported modulation of tDCS effects by neurotransmitter operations, and used motor-evoked potentials (MEPs) as outcome measurements. Consequently, they provided indirect measurements of neurotransmitters, which inherently limited the interpretation of results. In the present study, being an important factor in the introduction of a remote network effect, we focused on the change in neurotransmitter levels.

The present study directly measured changes in the extracellular dopamine level in the basal ganglia induced by tDCS using *in vivo* microdialysis in a rat model. This invasive procedure directly and locally measures compounds, such as extracellular dopamine, via a probe inserted into a target brain region (Navailles et al., 2004; Nitsche et al., 2006). Dopamine transmission in the striatum plays an essential role in the modulation of motor and cognitive symptoms caused by neurological disorders such as Parkinson's disease (PD) (Carlsson, 1972; Fahn, 2003), as well as in learning-induced neuroplasticity in both humans and rats (Adcock et al., 2006; Berridge, 2007; Rossato et al., 2009; Tanaka et al., 2011b). To understand the underlying mechanism of tDCS behavioral effects, we examined whether its application affected the dopaminergic systems in the striatum and showed that it had a direct and/or indirect effect.

## Materials and methods

The experimental protocol was approved by the Animal Care and Use Committee of the National Institute of Neuroscience (National Center of Neurology and Psychiatry, Tokyo, Japan). The experiments were conducted in accordance with the “Official Notification on Animal Experiments” (National Institute of Neuroscience, National Center of Neurology and Psychiatry notification no. 2010004, received 2010). Every effort was made to minimize the number of animals used in the experiments and their suffering.

### Animals

Male nine-week old Sprague Dawley rats (CLEA Japan, Inc., Tokyo, Japan) were housed at a temperature of 23 ± 1°C with a 12-h light/dark cycle (lights-on 08:00–20:00). Food and water were available *ad libitum*. Twenty-five rats were used for the microdialysis experiment, and 12 were used to investigate tissue damage.

### Microdialysis surgery

Twenty-five rats were divided into the following three groups: cathodal tDCS (*n* = 7), anodal (*n* = 7), and sham (*n* = 7). After at least 3 days of habituation to the animal colony, all rats were intraperitoneally (i.p.) anesthetized with a single shot of urethane (1 g/kg body weight) and placed in a stereotaxic apparatus. The skull was exposed and a small hole was made using a dental drill. A guide cannula (AG-6; Eicom Corporation, Kyoto, Japan) was implanted into the striatum [+1.0 mm anterior, +3.5 mm lateral, and −4.5 mm ventral to the bregma according to the stereotaxic atlas of Paxinos and Watson (1998)]. The guide cannula was fixed to the skull with resin dental cement.

### tDCS

**Figure 1:**
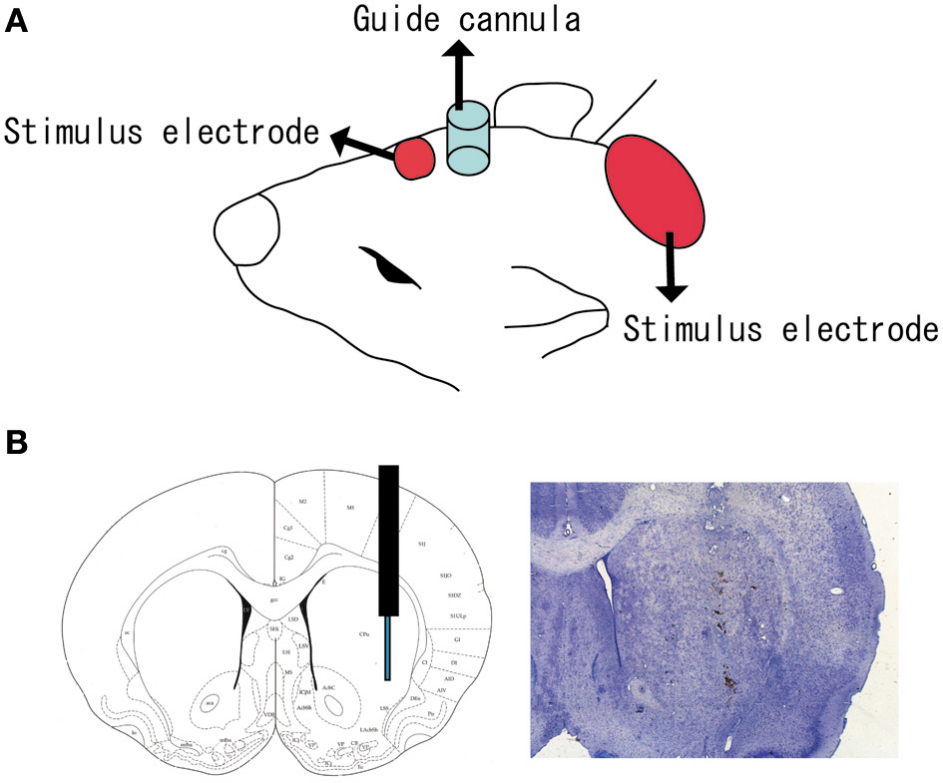
**Position of tDCS electrode and microdialysis probe. (A)** One electrode (small red square) of the stimulator was fixed to the skin with surgical tape just above the brain region including the cortex. The anatomical location corresponded to +2.0 to +7.0 mm anterior and +1.0 to +6.0 mm lateral to the bregma. A second electrode (large red ellipse) was placed on the neck. A guide cannula (blue cylinder) was fixed to the skull with resin dental cement; **(B)** a microdialysis probe was inserted into the striatum (+1.0 mm anterior, +3.5 mm lateral, and −4.5 mm ventral to the bregma).

The experimental tDCS setup was similar to that reported by Takano et al. (2010). One electrode of the stimulator (5 mm × 5 mm size) was fixed with surgical tape on the skin above the brain region including the cortex, and positioned in reference to the insertion point of the microdialysis guide cannula (Figure 1A) at an anatomical location roughly corresponding to +2.0 to +7.0 mm anterior and +1.0 to +6.0 mm lateral to the bregma (Paxinos and Watson, 1998). It was previously reported that the frontal cortex beneath the stimulation electrode had an anatomical connection to the striatum where the microdialysis probe was implanted (Ebrahimi et al., 1992; Gabbott et al., 2005). A second electrode was placed on the animal's neck in a similar way.

Cathodal or anodal tDCS was applied continuously for 10 min with a current intensity of 800 μA from the scalp electrode using a DC stimulator (STG1002; Multi Channel Systems, Germany). Cathodal stimulation was applied for 10 s to the sham group with a current intensity of 10 μA from the scalp electrode. The safety limit of the stimulator was 120 V. The current intensity of 800 μA, corresponding to a current density of 32.0 A/m^2^ in the present setting, was used to maximize the effects of tDCS within the safety limits reported in a previous rat tDCS study using cathodal stimulation (Liebetanz et al., 2009), although the safety limits of anodal stimulation was not specified.

### Dopamine and serotonin measurements

Stimulation and *in vivo* microdialysis were carried out under urethane anesthesia. Body temperature and heart rate were monitored and remained at approximately 37°C and 350 bpm, respectively. No remarkable changes in vigilance were observed by visual inspection of body movement. After surgery, microdialysis probes (AI-6-02; 2-mm membrane length; Eicom Corporation) were slowly inserted into the striatum through the guide cannula (Figure 1B). The probe was perfused continuously at a flow rate of 2 μ l/min with artificial cerebrospinal fluid (aCSF) containing 0.9 mM MgCl_2_, 147.0 mM NaCl, 4.0 mM KCl, and 1.2 mM CaCl_2_. The collection and analysis of perfusion solution were performed at 10 min intervals over the duration of the experiment. The concentrations were judged stable when the fluctuation range of extracellular dopamine and serotonin levels did not exceed ±5% during six consecutive fractions and when there was not unidirectional fluctuation over more than three fractions (Alex et al., 2005; Kitaichi et al., 2010). After confirming the stability, three fractions were collected from the rat striatum before the application of tDCS as a baseline. Following tDCS, dialysis samples were collected every 10 min until at least 400 min after the onset of stimulation.

Dialysis fractions were analyzed using high-performance liquid chromatography (HPLC) with an electrochemical detection (ECD) system (Eicom Corporation). The extracellular serotonin level in the striatum was also measured, as it is known to be associated with the dopamine level (Di Giovanni et al., 2008; Navailles and De Deurwaerdere, 2011). Dopamine and serotonin were separated by a column with a mobile phase containing 99% 0.1 M sodium phosphate buffer (pH 6.0), 500 mg/L sodium 1-decanesulfonate, 50 mg/L ethylenediaminetetraacetic acid disodium salt (EDTA-2Na), and 1% methanol. The mobile phase was delivered at a flow rate of 500 μ l/min. Before every experiment dopamine hydrochloride and serotonin hydrochloride, as the standard reagents, were dissolved in solvent and injected into the HPLC system. Retention times of dopamine and serotonin were calculated from the peaks detected in the chromatograph. During each experiment, the retention time of substances, which were collected from the striatum, was calculated. When the measured retention time matched with those of the standard reagents, collected substances were judged as dopamine or serotonin. All the measurement conditions such as mobile phase for calibration were same as those for actual measurements of rats. Dopamine and serotonin were quantified by calculating the peak areas (Alex et al., 2005; Kitaichi et al., 2010).

To confirm the insertion position of the microdialysis probe after completion of the experiment, all rats were deeply anesthetized with sodium pentobarbital (50 mg/kg body weight, i.p.) and perfused through the heart sequentially with 1 × phosphate buffered saline (PBS) followed by 10% formalin neutral buffer solution. Rat brains were post-fixed and sucrose-substituted at 4°C, and 20−μm-thick coronal sections were cut through the striatum (−1 mm to 3 mm anterior to the bregma) on a cryostat. These were then thaw-mounted on 3-aminopropyltriethoxysilane (APTS)-coated slides and stained with cresyl violet using standard procedures.

### tDCS-induced tissue damage

Histological examination was performed to determine the effects of tDCS on the brain tissue and skin beneath the scalp electrode. At 24 h after the application of tDCS, 12 rats (cathodal tDCS and sham groups; *n* = 6 per group) were deeply anesthetized with sodium pentobarbital (50 mg/kg body weight, i.p.), and 10−μ m-thick coronal sections of their brains were prepared and stained with cresyl violet using a method similar to that described above. The skins were post-fixed at 4°C, dehydrated with ethanol and xylene, and paraffin-embedded. Sections (5−μ m thick) of rat skin from just below the scalp electrode were cut on a microtome, thaw-mounted on APTS-coated slides, and stained with hematoxylin and eosin (HE) using standard procedures. Morphological changes were then evaluated. To confirm whether tDCS leads to apoptosis, 10−μ m-thick coronal sections were prepared from the same animals and stained with terminal deoxynucleotidyl transferase-mediated biotinylated UTP nick-end labeling (TUNEL) (Kobayashi et al., 2004). To minimize the number of animals used in the experiments, we omitted the anodal tDCS group, which showed no significant changes in dopamine or serotonin levels in the microdialysis experiment (see section Results).

### Statistical analysis

Data from four rats in which a probe had not been accurately inserted into the striatum (Figure 1B) were excluded from statistical analysis. Extracellular dopamine and serotonin levels were expressed as percentage signal changes from baseline values before tDCS application. Group data are presented as mean ± standard error (*SEM*). The statistical significance of differences between groups was assessed by repeated measures analysis of variance (ANOVA) with time (TIME) as a within-subject factor and group (GROUP) as a between-subject factor. This was followed by the *post-hoc* Bonferroni test using SPSS software (SPSS Inc., Chicago, IL). To investigate whether the time effect differed between groups, we confirmed the “TIME” × “GROUP” interaction. *P*-values less than 5% were considered statistically significant. To investigate when the change in dopamine level became prominent, data at each time point were compared with the baseline using the paired *t*-tests. For the same purpose, data from the tDCS group were compared with those in the sham group at each time point separately using the unpaired two-sample *t*-tests.

## Results

**Figure 2:**
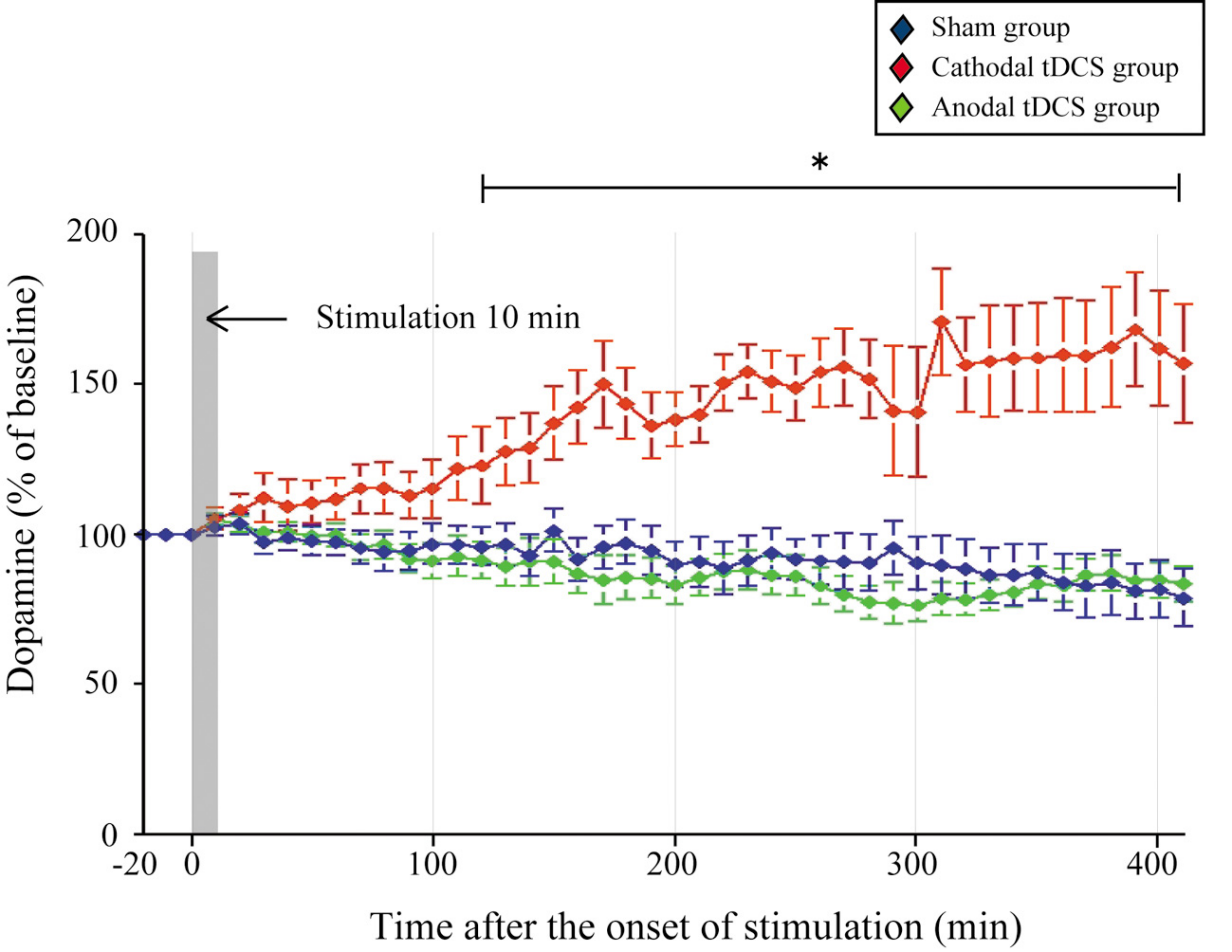
**Effect of tDCS on extracellular dopamine levels in the striatum.** The absolute basal dialysis levels of dopamine in the striatum detected 10 min before the interventions did not differ between groups. Dopamine levels were expressed as percentage signal changes from baseline values before the tDCS application. Group data are presented as the mean ± *SEM*. Cathodal, but not anodal, tDCS significantly increased extracellular dopamine levels in the striatum. ^*^p < 0.001.

Statistical analysis was carried out on rats in the cathodal tDCS (*n* = 7), anodal tDCS (*n* = 7), and sham (*n* = 7) groups. To examine whether cathodal or anodal tDCS affected extracellular dopamine and serotonin levels in the striatum, we investigated the effect of tDCS using *in vivo* microdialysis. The absolute basal dialysis levels of dopamine and serotonin detected 10 min before the interventions did not differ between the three groups [Table 1; dopamine, *F*_(2, 20)_ = 0.389, *p* = 0.684; serotonin, *F*_(2, 20)_ = 0.242, *p* = 0.788]. These basal levels compared well with the previous studies (Baumann et al., 2008; Kitaichi et al., 2010). Following the application of cathodal tDCS for 10 min, the extracellular dopamine levels continuously increased and this effect lasted for more than 400 min after the stimulation ceased (Figure 2).

**Table 1:** **Absolute basal dialysis levels of dopamine and serotonin**.

**Group**	**Dopamine (nM)**	**Serotonin (nM)**
Sham	0.61 ± 0.070	0.11 ± 0.076
Cathodal tDCS	0.62 ± 0.076	0.11 ± 0.062
Anodal tDCS	0.69 ± 0.071	0.06 ± 0.012

Data are presented as the mean ± SEM.

The dopamine time-course in the striatum differed significantly between the three groups [main effect of GROUP, *F*_(1, 20)_ = 27.386, *p* < 0.001; main effect of TIME, *F*_(44, 880)_= 0.514, *p* = 0.997; interaction of GROUP × TIME, *F*_(2, 88)_ = 4.674, *p* < 0.001]. The dopamine increases in the cathodal tDCS group were significantly greater than those in the sham and the anodal tDCS groups (*p* < 0.001 for both, *post-hoc* test). These increases became statistically prominent 120 min after the stimulation compared with the pre-intervention period (*p* < 0.05, paired *t*-test), and lasted throughout the observation period. Furthermore, the dopamine increases in the cathodal tDCS group were significantly greater than those in the sham group from 120 min after stimulation (*p* < 0.05, unpaired two-sample *t*-test). By contrast, the application of anodal tDCS did not induce significant increases in extracellular dopamine levels.

**Figure 3:**
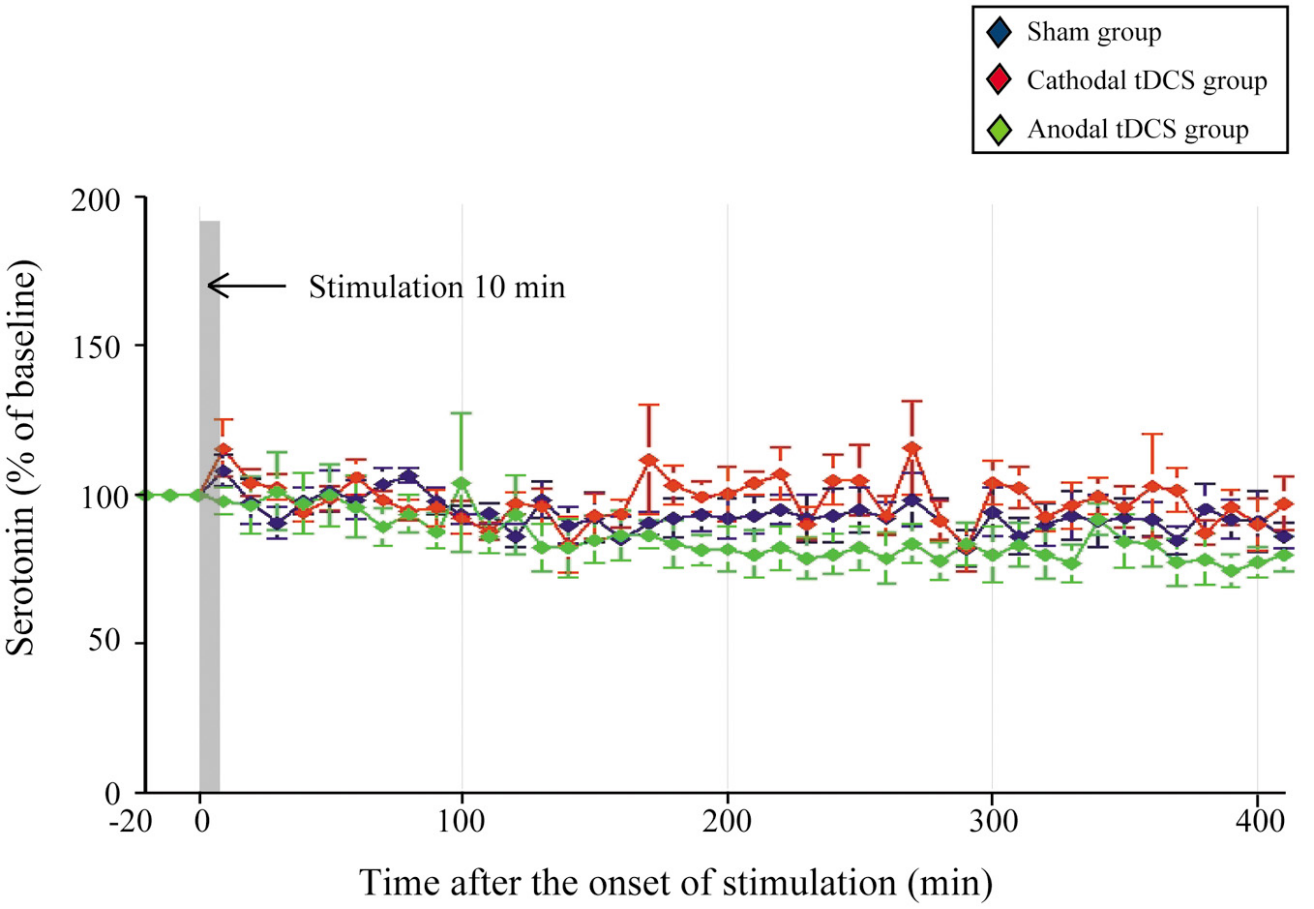
**Effect of tDCS on extracellular serotonin levels in the striatum.** The absolute basal dialysis levels of serotonin in the striatum detected 10 min before the interventions did not differ between groups. Serotonin levels were expressed as percentage signal changes from baseline values before the tDCS application. Group data are presented as the mean ± *SEM*. None of the tDCS applications significantly affected extracellular serotonin levels.

There were no significant changes in extracellular serotonin levels in the striatum in any group, indicating that they were unaffected by the application of either cathodal or anodal tDCS [Figure 3; main effect of GROUP, *F*_(1, 20)_ = 2.016, *p* = 0.159; interaction of GROUP × TIME, *F*_(2, 86)_ = 0.997, *p* = 0.540, respectively].

**Figure 4:**
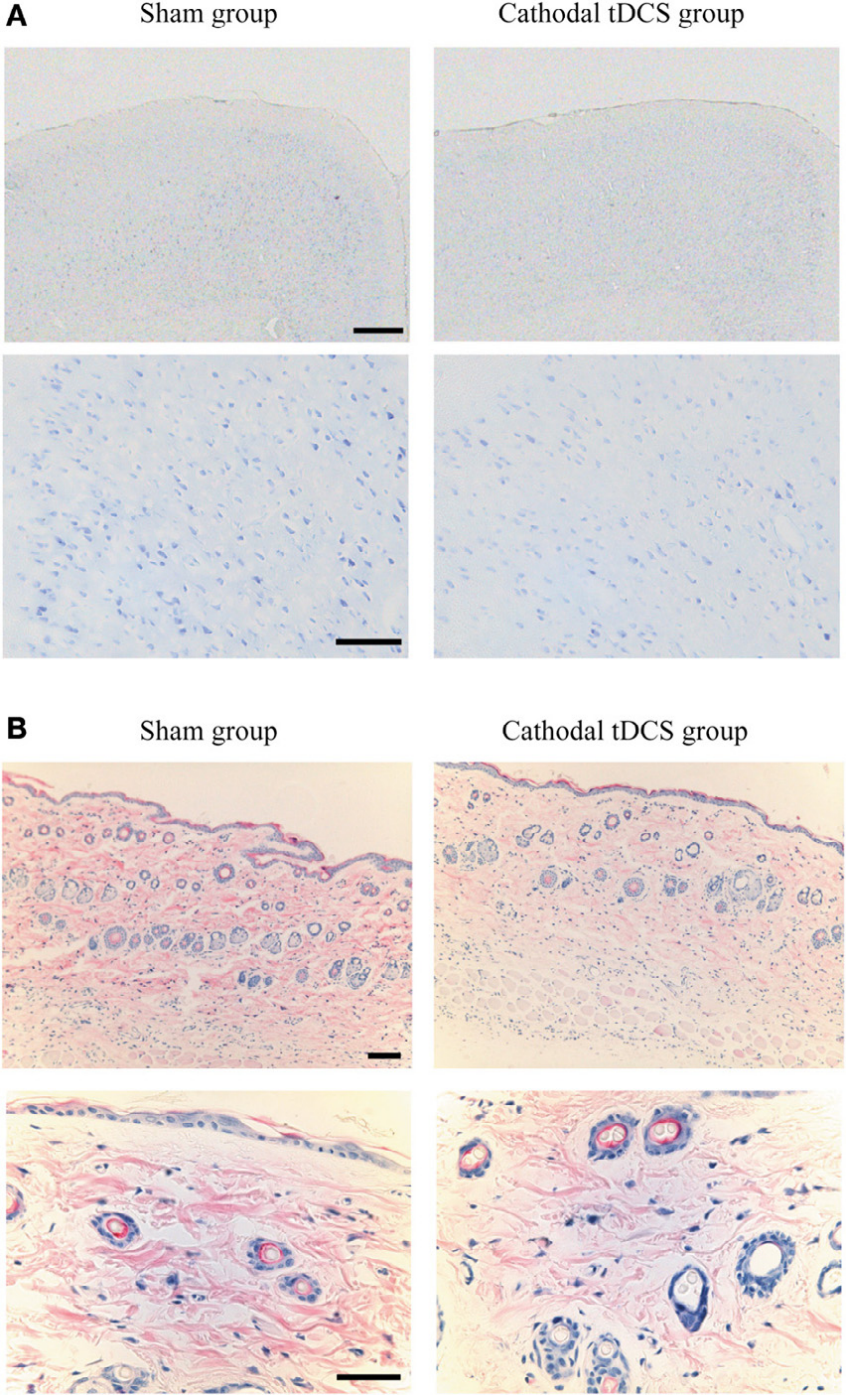
**Histological examination following tDCS. (A)** Cresyl violet staining of the brain tissue below the scalp electrode in the sham group (left) and the cathodal tDCS group (right) 24 h after tDCS application. Scale bars: 1 mm for upper panels, 100 μm for lower panels; **(B)** HE staining of the skin below the scalp electrode in the sham group (left) and the cathodal tDCS group (right) 24 h after tDCS application. Scale bars: 100 μm for upper panels, 50 μm for lower panels. No morphological change was observed in brain tissue and skin.

One day after the application of tDCS, tissues were dissected and processed for sectioning. The sections through the cortex were used for histological examination with cresyl violet staining. No abnormal findings regarding cellular morphology, such as chromatolysis or atrophied tissue, were observed in the cortex below the scalp electrode of rats in either the cathodal tDCS group or the sham group (Figure 4A). No apoptosis was found in either group (data not shown). The sections through the skin below the scalp electrode were analyzed by HE staining, revealing no abnormalities such as dead tissue, inclusion, or multicell spheroids in either group (Figure 4B).

## Discussion

The main finding of the present study was that cathodal tDCS induced a significant increase in extracellular dopamine levels in the rat striatum, whereas anodal tDCS in the same area had no effect. Although recent magnetic-resonance spectroscopy studies have shown direct effects of tDCS on cortical GABA concentration (Stagg et al., 2009), the effect of tDCS on subcortical neurotransmitters has remained unclear. The present results suggest that tDCS has a direct and/or indirect effect on the dopaminergic system in the basal ganglia. The finding that tDCS affected dopamine but not serotonin is consistent with a previous study that reported no changes in the rat serotonergic system following repetitive transcranial magnetic stimulation (rTMS) (Kanno et al., 2004). As the tDCS methods used here did not damage tissue in the cortex or the skin below the scalp electrode, it is unlikely that the rise in dopamine was induced by non-specific cellular injury.

### Interpretation of the cathodal tDCS effect

Several studies have shown that diverse areas of the cerebral cortex, including the sensory, motor, and associated regions, project to the subcortical regions, including the striatum (McGeorge and Faull, 1989; Lang et al., 2005; Halko et al., 2011). Furthermore, it has been reported that descending pathways from the frontal cortex to the striatum modulate a release of dopamine in subcortical areas in both animal and human experiments (Murase et al., 1993; Taber and Fibiger, 1995; Karreman and Moghaddam, 1996). Considering that tDCS has been found to improve symptoms of PD in both animal and human experiments (Boggio et al., 2006; Fregni et al., 2006; Benninger et al., 2010; Gruner et al., 2010; Li et al., 2011), it is plausible that tDCS could directly and/or indirectly affect the extracellular dopamine levels in the striatum.

However, our finding that cathodal tDCS, but not anodal, increased extracellular dopamine levels is contradictory to our prediction and inconsistent with previous studies. For example, cathodal tDCS has been thought to induce suppression of motor function and learning by inhibiting neuronal excitability of the cortex (Murphy et al., 2009; Nitsche et al., 2009; Bachmann et al., 2010; Benninger et al., 2010; Fox, 2011; Schambra et al., 2011; Tanaka et al., 2011a). In addition, rTMS has been suggested to induce increased extracellular dopamine levels in the striatum by facilitating neuronal excitability of the cortex (Keck et al., 2002; Strafella et al., 2003; Ohnishi et al., 2004). We acknowledge that the present finding is contradictory to such studies.

To our knowledge, this is the first report that directly measured extracellular dopamine levels in the striatum-induced by tDCS. Although the mechanism underlying the long-lasting effect of cathodal tDCS observed in the present study has not yet been determined, we offer the following speculations. It has been widely accepted that cathodal tDCS decreases cortical neuronal activity (Bindman et al., 1964; Purpura and McMurtry, 1965; Fritsch et al., 2010). Meanwhile, a recent report showed that cathodal, but not anodal, tDCS induced paired pulse facilitation in the somatosensory cortex of rabbits following stimulation of the ventroposterior medial thalamic nucleus (Marquez-Ruiz et al., 2012). This study suggests that cathodal tDCS may specifically facilitate synaptic plasticity. Furthermore, cathodal, but not anodal, tDCS was shown to facilitate working memory and skill learning 21 days after treatment, implying that cathodal tDCS might facilitate long-term homeostatic cortical metaplasticity (Dockery et al., 2011). Although the cathodal tDCS may instantaneously decrease neuronal activity beneath the stimulus electrode, these findings suggest that cathodal tDCS may specifically induce a long-term plastic change thorough some metabolic changes, such as BDNF release (Fritsch et al., 2010). We speculate that the increase of dopamine release in the striatum may contribute to such long-term plasticity.

Alternatively, the cortico-basal ganglia neural circuit contains both excitatory and inhibitory projections (Alexander and Crutcher, 1990; Nambu et al., 2000) in an intrinsic neuronal network. Therefore, we must also consider the possibility that cathodal tDCS affects extracellular dopamine levels in the striatum through an inhibitory circuit.

### Experimental limitations

General anesthesia affects brain metabolism, neuronal activity, and response to sensory stimuli (Buzsaki et al., 1983; Friedberg et al., 1999); it is thus conceivable that the present results were partly influenced by anesthesia. However, this is unlikely to be the only reason for the cathodal tDCS-specific dopamine increase, as the anesthetic procedure was common to all stimulation conditions. Moreover, in a previous study, alteration of extracellular dopamine levels in the striatum induced by medial forebrain bundle stimulation did not differ between urethane-anesthetized and awake animals (Tepper et al., 1991).

The contamination effect induced by the tDCS reference electrode on the neck should be considered. Although the electrode-current density in this region was lower and less effective, and there is no brain tissue beneath the tDCS reference electrode on the neck, it was unclear exactly how the current flowed between the two electrodes in the present experimental setup. Furthermore, the current delivered by the stimulation electrode might have reached the striatum through the guide cannula, even though the dental cement used to fix it was an electrical insulator. To rule out this possibility, extracellular dopamine levels should be measured using a non-invasive technique such as positron-emission tomography.

Though polarity of their affective stimulation electrode differed from that of the present study, some reports have shown that tDCS improves PD symptoms in both animals and humans (Boggio et al., 2006; Fregni et al., 2006; Benninger et al., 2010; Gruner et al., 2010; Li et al., 2011). An underlying mechanism of such clinical effects could be an increase in extracellular dopamine levels in the striatum induced by tDCS. However, it is as of yet unclear whether 150% of basal dopamine level is clinically relevant and if tDCS will increase dopamine levels in patients whose dopaminergic neurons have degenerated. Therefore, we recommend caution in correlating the present finding to clinical application of tDCS.

In conclusion, we demonstrated that cathodal tDCS increased extracellular dopamine levels in the rat striatum. This suggests that tDCS has a direct and/or indirect effect on the dopaminergic system in the subcortical area. Further work to determine the mechanism underlying tDCS effects on cortical-basal ganglia functions could benefit our understanding of learning-induced neuroplasticity and the development of new clinical interventions.

## Author contributions

Tomoko Tanaka, Yuji Takano, Satoshi Tanaka, Naoyuki Hironaka, Kazuto Kobayashi, Takashi Hanakawa, Katsumi Watanabe, and Manabu Honda designed the research; Tomoko Tanaka performed the research; Tomoko Tanaka analyzed the data; Tomoko Tanaka, Satoshi Tanaka, and Manabu Honda wrote the paper.

### Conflict of interest statement

The authors declare that the research was conducted in the absence of any commercial or financial relationships that could be construed as a potential conflict of interest.
